# Modulating G-quadruplexes for therapeutic intervention: Structural diversity, stability, and emerging nucleic-acid-based strategies

**DOI:** 10.1016/j.omtn.2026.102906

**Published:** 2026-03-19

**Authors:** Mariia Sokulska, Maria Nalewaj, Tomasz Czapik, Marta Rachwalak, Marta Szabat

**Affiliations:** 1 Institute of Bioorganic Chemistry, Polish Academy of Sciences, Noskowskiego 12/14, 61-704 Poznan, Poland; 2 Biomolecular Division of Biomolecular and Cellular Medicine, Department of Laboratory Medicine, Karolinska Institutet, ANA Futura, Alfred-Nobels-Allé 8, 14152 Huddinge, Sweden; 3 Karolinska ATMP Center, ANA Futura, 14152 Huddinge, Sweden

**Keywords:** MT: oligonucleotides: therapies and applications, RNA/DNA G-quadruplex, G-quadruplex stability, antisense oligonucleotides, peptide nucleic acids, conjugates, G4-specific ligands, viral genome, therapeutic tools

## Abstract

G-quadruplexes (G4s) are non-canonical nucleic acid structures formed by guanine-rich DNA or RNA sequences. The G4s are formed by planar G-tetrads and stabilized by monovalent metal cations such as potassium or sodium. The structural diversity of G4s arises from differences in strand orientation, loop arrangement, and molecularity, leading to multiple topologies. The length of guanine tracts (G-tracts) and the number of strands involved further influence G4 folding, stability, and biological function. G-quadruplexes are commonly found in key genomic regions such as telomeres, promoters, and untranslated regions, where they play important roles in regulating fundamental biological processes, including replication, transcription, and translation. In this review, we summarize the molecular factors influencing G4 formation and stability. We also discuss recent advances in therapeutic strategies for targeting G-quadruplexes. Particular attention is given to antisense oligonucleotides (ASOs), ASO-ligand conjugates, peptide nucleic acids (PNAs), and PNA-conjugates as chemical tools for selective G4 recognition and modulation. We highlight the potential of nucleic-acid-based approaches for molecular therapeutics, including applications in anticancer and antiviral treatments.

## Graphical abstract

**Figure:**
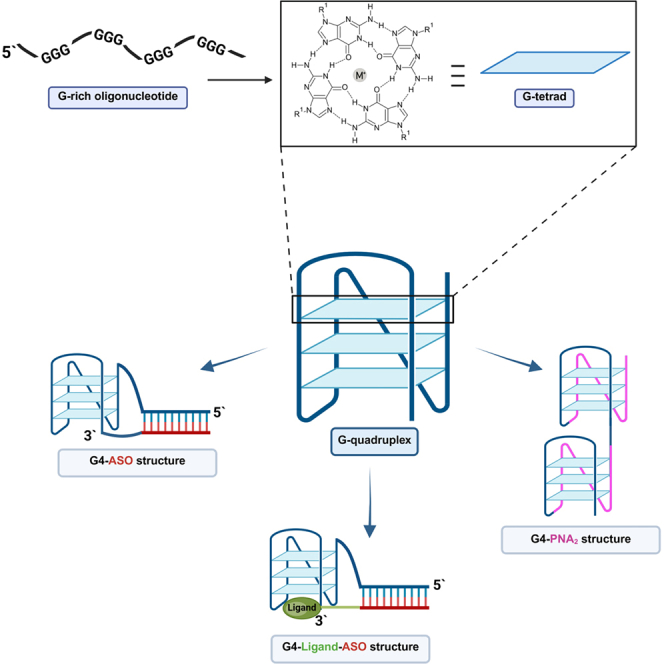

## Teaser

This review explains how sequence features, structural polymorphism, and molecular context govern the formation, stability, and biological roles of G-quadruplexes. It emphasizes recent progress in G4 selective targeting using nucleic-acid-based tools, underscoring their growing potential as precise therapeutic strategies in cancer treatment and antiviral applications.

## Introduction

Guanine-rich sequences within DNA and RNA can adopt non-canonical four-stranded structures known as G-quadruplexes (G4s). These structures arise from the stacking of planar G-tetrads, in which four guanine bases are held together through Hoogsteen hydrogen bonding and stabilized by monovalent metal cations, particularly potassium (K^+^) and sodium (Na^+^). G-quadruplexes have been previously identified in functionally relevant genomic regions such as telomeres, gene promoters, and untranslated regions (UTRs). Furthermore, G4 structures are involved in diverse biological processes, including replication, transcription, translation, and genome stability. Their regulatory potential, along with emerging applications in therapy and nanotechnology, has been widely studied in recent years.^1^^,^^2^^,^^3^^,^^4^

The structural polymorphism of G-quadruplexes arises from several intrinsic features, notably strand orientation, glycosidic bond conformation, loop configuration, and the number of strands participating in G-tetrad formation. G4 topologies are broadly categorized as parallel, antiparallel, or hybrid (mixed). In parallel structures, all four strands run in the same 5′–3′ direction; antiparallel G4s feature two strands oriented in the opposite direction to the other two, whereas hybrid structures contain a mix of strand polarities. These orientations are tightly coupled to the glycosidic bond conformations of the guanine nucleosides, which can adopt either a *syn* or *anti* configuration. The arrangement of these conformations, referred to as step patterns, plays a pivotal role in determining the thermodynamic stability of the G-quadruplex.

It is known that the length of guanine tracts (G-tracts) within a sequence further contributes to folding propensity and structural variability.^5^^,^^6^ The relationship between G-tract length and G-quadruplex (G4) polymorphism is governed by register availability, loop architecture, strand orientation, and environmental conditions rather than by *syn-anti* glycosidic uniformity alone. Sequences containing G2 tracts rarely form stable G4 structures, as two guanines per tract are generally insufficient to support stable tetrad stacking. Consequently, their limited polymorphism primarily reflects poor thermodynamic stability rather than restricted glycosidic conformations. G3 tracts provide sufficient stability for G4 formation, and sequences containing G3 tracts exhibit high polymorphism. This feature is driven by the interplay of loop length and composition, loop arrangement, cation type, and sequence context. Additional factors, such as flanking sequences, further modulate the conformational landscape, leading to multiple coexisting G4 topologies under different environmental conditions. In contrast, longer G-tracts (e.g., G4) can introduce multiple-strand registers, which in some cases increase conformational heterogeneity rather than constrain it.^5^^,^^6^^,^^7^^,^^8^ Moreover, G-quadruplexes can form a variety of topologies, including unimolecular, bimolecular, and tetramolecular complexes. The number of strands involved affects the overall topology, folding kinetics, and biological function of the G4. Although tetramolecular G-quadruplexes have been extensively characterized *in vitro*^9^^,^^10^ and provide valuable structural insights, they are generally formed under non-physiological conditions and therefore not considered the predominant G4 species *in vivo*. Current evidence supports unimolecular G4s, and in certain contexts, bimolecular G4s as the biologically relevant forms, consistent with intracellular strand availability and genomic organization.^11^^,^^12^^,^^13^ Overall, the molecularity of G-quadruplexes impacts their stability and functional roles in biological and structural contexts.

In this review, we focus on G4 structures and the diverse factors that influence their formation and stability. These include sequence composition, loop length, the presence of monovalent cations, the incorporation of chemical modifications, and the G4-specific ligands. We then explore therapeutic strategies targeting G-quadruplex structures, with a particular emphasis on antisense oligonucleotides (ASOs). In addition to conventional ASO approaches, we discuss more advanced ASO-ligand conjugates designed to enhance G4 binding specificity and biological activity. Furthermore, we highlight the use of peptide nucleic acids (PNAs) and PNA conjugates as versatile tools for G4 targeting, offering improved stability and affinity over traditional oligonucleotide-based strategies. Finally, other recently reported strategies for selective G4 targeting are briefly discussed.

By integrating current knowledge on G4s biology and targeting methodologies, this review aims to provide a comprehensive overview of the molecular determinants of G4 stability and the innovative nucleic-acid-based tools developed to modulate G4 function for potential therapeutic applications, with particular emphasis on G4 targets identified within viral genomes.

## Examples of factors that contribute to the G-quadruplex formation and stability

In general, G-quadruplexes can be extremely stable, although their topology and stability depend on many factors. RNA G-quadruplexes generally exhibit greater stability than their DNA counterparts under a wide range of conditions.^14^^,^^15^ This enhanced stability is primarily due to the presence of the 2′-hydroxyl group of ribose, which forces the C3′-endo sugar pucker and promotes a rigid backbone geometry. Consequently, this feature strongly favors parallel G-quadruplex topologies.^16^^,^^17^ The resulting structural uniformity reduces flexibility and conformational polymorphism, while hydration effects further enhance stability. In parallel RNA G4s, the ordered geometry supports a homogeneous network of water molecules within the grooves, whereas antiparallel G-quadruplexes show a less ordered hydration pattern.^18^

**Figure 1:**
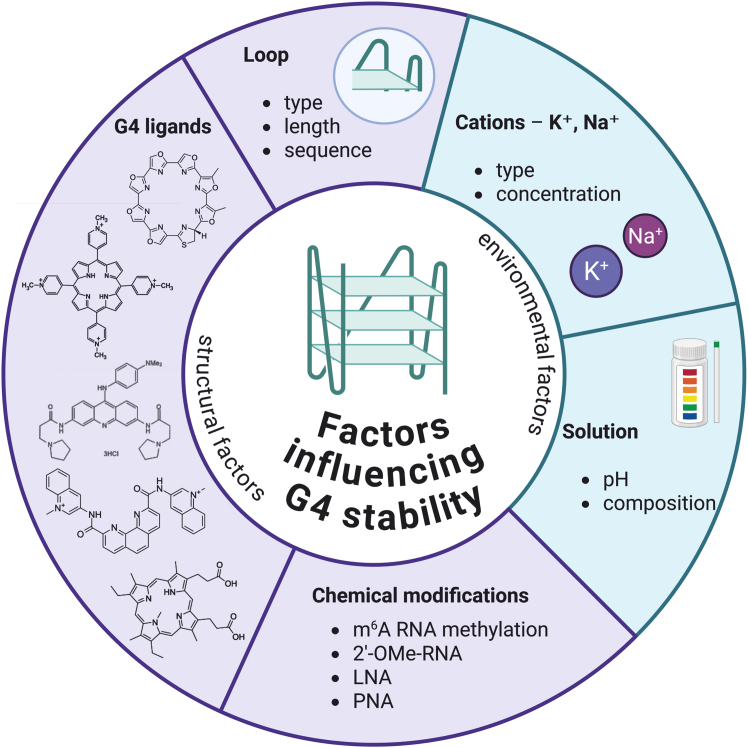
A schematic illustration of the key factors influencing the G4s formation and stability

Overall, the stability of G-quadruplexes is influenced by factors such as the loop type, length, and sequence,^19^ the type and concentration of cations present in the solution,^20^ and the composition and pH of the solution.^21^ Furthermore, the presence of chemical modifications within the nucleotides forming G4^22^ or the presence of G4-specific ligands^23^ has an important influence on the G-quadruplex conformation. In the following sections, we focus on selected structural and environmental factors that affect the formation and stability of G4s, which are summarized in Figure 1.

## The effect of loop sequence and length on G-quadruplex formation

The loops connecting G-tetrads consist of various nucleotides. They vary in length and sequence, affecting the overall conformation and stability of the G-quadruplex structure. The loop length is one of the most important factors determining the G4s folding. Parallel unimolecular or bimolecular G-quadruplexes, both RNA and DNA, have only external loops. Studies of the relationship between loop length and thermodynamic stability of unimolecular, parallel RNA G4s indicate that stability decreases with increasing loop length.^24^^,^^25^ For instance, Pandey et al. reported that RNA G-quadruplexes consisting of two G-tetrads are stable (melting temperature 60°C) only when their loops are single-nucleotide in length (G2UxG2UxG2UxG2: x = 1). Extending the loops to 3 or 7 residues decreases their melting temperatures to 30°C and 19°C, respectively.^26^ Moreover, thermodynamic stability and folding topology of DNA G4s are also affected by the loop length and its arrangement.^27^ Long loops in an antiparallel DNA G4 structure enhance its stability, possibly due to secondary interactions in the loops or interactions between loops and the G-tetrads.^28^^,^^29^ The folding properties and stability of intramolecular G-quadruplexes are strongly influenced by loop length. Three single-residue loops enforce a parallel-stranded topology. Longer loops, or combinations of short and long loops, favor both parallel and antiparallel folding topologies. In such cases, multiple conformations with similar free energies are likely to coexist in solution.^28^ Furthermore, long-loop G-quadruplexes are generally less stable than conventional G4s, but their stability increases when the loops form hairpins. Hairpin-G4s are biologically relevant structures that form stably within the genome and regulate gene expression. This study expands our understanding of G4 diversity and highlights the functional importance of hairpin-containing G4s in the human genome.^30^

The formation of G-quadruplexes depends not only on the loop length but also on the nucleotide composition of the loops. Changing even a single nucleotide residue in the loop region can affect the structure and stability of the G-quadruplex. For instance, Fox and co-workers examined properties of intramolecular G-quadruplexes with G3 tracts separated by single-base loops.^31^ The authors found that G4s with single adenine loops exhibited an approximate 8°C decrease in stability for each T-to-A substitution.^31^ Notable studies by Chang and colleagues^13^ investigated the effects of loop length and base composition on G-quadruplex formation in (G_3_HG_3_N_m_G_3_HG_3_) sequences with and without flanking nucleotides, where H is T, A, or C; N is T, A, C, or G; and m is the number of nucleotides.^32^ They found that (G_3_HG_3_N_m_G_3_HG_3_) sequences can fold into multimeric G4 structures, while (G_3_HG_3_N_m_G_3_HG_3_) with flanking nucleotides tend to form monomeric G-quadruplexes.^32^ Additionally, for the (G_3_HG_3_N_m_G_3_HG_3_) sequence, increasing the length of the central loop generally results in a lower melting temperature of adenine. In contrast, loops containing a single thymidine base exhibit higher thermal stability than those containing a single adenine base.^31^^,^^32^

Recently, Maiti and co-workers revealed that cytosine-containing RNA G4s are less stable (melting temperature 43°C) than G-quadruplexes with adenosine or uridine residues within the loop (melting temperature 70°C or 60°C, respectively).^26^ In contrast, for DNA G4 structures, adenine is significantly disfavored over the thymine and cytosine bases when located at the first position of the central loop. Additionally, the positional effect of cytosine residues in the loop of DNA G4s depends on the presence of cations.^14^^,^^15^

In addition to loop length and nucleotide composition, recent advances in high-resolution structural studies have revealed that specific spatial loop conformations can significantly influence G4 stability and ligand recognition. For instance, a 3′-end snap-back loop configuration has been shown to fold back onto the terminal G-tetrad. This conformation partially occludes the external binding surface, thereby hindering ligand accessibility and stacking interactions on that terminal tetrad.^33^^,^^34^^,^^35^ Similarly, G4s containing bulge-associated loop elements exhibit distinct topological and dynamic characteristics. These features may influence overall stability and generate unique molecular recognition interfaces.^36^ Recently, structural insights into the bulge-containing KRAS oncogene promoter G4 in complex with berberine and coptisine were obtained by NMR analysis.^36^ The resolved structures revealed a 2:1 ligand binding mode. This interaction was characterized by a “quasi-triad plane” that stacks over the two external G-tetrads. The binding involves both π-stacking and electrostatic interactions.^36^ Together, these findings suggest that, beyond loop length and sequence composition, higher-order loop architectures should also be considered as important factors that affect the G4 stability and targeting specificity. Stable G-quadruplexes can also form when nucleotide residues in the loops are replaced with non-nucleotide linkers, e.g., residues without heterocyclic bases (abasic), or aliphatic linkers. For example, incorporation of an abasic residue into the loop can increase the conformational freedom of the resulting G4 more than when the loop contains thymidine, cytidine, or adenosine residues.^31^^,^^37^ For instance, Esposito et al. investigated the effects of abasic sites on structural, thermodynamic, and kinetic properties of G-quadruplexes.^37^ The authors found that all studied sequences preserve their ability to fold into G4s. However, the experiments showed the sequence-dependent effects on the G4 structural flexibility and stability.^37^

Furthermore, the presence of aliphatic linkers in the loop sequence of G-rich oligonucleotides can promote the formation of a parallel G-quadruplex structure. It was previously described that the presence of abasic residue and long aliphatic linkers in the loop promotes and stabilizes the formation of a parallel G-quadruplex.^38^^,^^39^ Early studies by Risitano and Fox demonstrated that replacing the TTA loops of an intramolecular DNA G4 with non-nucleosidic aliphatic linkers, such as propanediol, hexaethylene glycol, or octanediol, induces a conformational transition from an antiparallel to a parallel topology.^38^ They found that DNA G4 stability increases in the order propanediol < hexaethylene glycol < octanediol.^38^

Similar conclusions were drawn from later studies by Virgilio et al., who investigated the antiproliferative properties of the aptamer d(GGGT)_4_ and its analogues containing abasic-site mimic loops.^40^ Their results showed that the modified oligonucleotides adopt structures highly similar to the dimeric parallel G4 of the parent aptamer. No significant change in biological stability was observed, as indicated by nuclease resistance assays.^40^ Collectively, these findings indicate that abasic residues or flexible aliphatic linkers in loop positions do not necessarily disrupt G-quadruplex formation. They can be fully compatible with stable parallel topologies, depending on the sequence context and loop design. Mechanistically, this behavior results from reduced steric constraints and the elimination of base-specific interactions that otherwise favor alternative folding pathways. By increasing conformational freedom and disfavoring loop–tetrad or loop–loop hydrogen bonding, abasic sites and alkyl spacers stabilize compact parallel G4 structures primarily through entropic and geometric effects rather than direct stacking interactions.^38^^,^^41^

Furthermore, structure-activity relationship studies of G4-binding ligands consistently show that removal of the aromatic core abolishes binding. Deletion or modification of aliphatic side chains results only in moderate reductions in affinity or selectivity, rather than complete loss of binding.^42^^,^^43^^,^^44^^,^^45^ Thus, unlike abasic loop modifications in oligonucleotides, aliphatic side chains in small molecules do not independently drive G4 topology or folding.

## The effect of cations on G-quadruplexes formation

Sodium and potassium are physiologically important cations; therefore, most research focuses on understanding their influence on G-quadruplex structure and stability. Overall, potassium and sodium cations stabilize G4s by their binding within the central channel of the G-core, reducing repulsion between guanine carbonyl oxygen atoms and promoting stacking interactions of G-quartets. Nevertheless, other monovalent and divalent cations have also been shown to influence the folding of G-quadruplexes. The type of cations not only determines the stability but can also induce structural polymorphism of G4 structures.^20^^,^^46^

In G-quadruplex structures, ions can be positioned in or between the planes of the G-quartets. Monovalent cations such as Na^+^, NH_4_^+^, or K^+^ are located in the central channel, where they neutralize the negative charge accumulated through the carbonyl oxygen O6 atoms. Cations can also interact with other groups of atoms located in the loops and grooves. The ionic radius is one of the major factors in cation selection for G-quadruplex studies. The potassium atom coordinates the eight carbonyl oxygen atoms of adjacent tetrads, thus strongly stabilizing the guanine tetrads. The smaller cation, Na^+^, is sufficiently small to coordinate either within the plane of the guanosine tetrad or, like K^+^, between the two planes. Due to the smaller ionic radius of the sodium cation, the G-tetrad stabilizing effect is significantly weaker.^20^^,^^47^^,^^48^ Beyond ionic radius, the coordination properties and geometry of cations strongly affect G4 stability.^49^^,^^50^ The cation geometry influences how effectively they interact with carbonyl oxygens and stabilize tetrad stacking. Both the size and shape of the cation relative to the G4 channel are important. A recent biophysical study reviews how different cations affect G4 folding and stability. It emphasizes that G-quadruplex stability is primarily determined by specific coordination interactions between cations and the carbonyl oxygens of guanine tetrads, rather than by ionic size alone.^50^

Moreover, Na^+^ preferentially stabilizes antiparallel G-quadruplex structures, whereas K^+^ generally favors parallel or hybrid G4 topologies, thereby shifting the conformational equilibrium to disfavor antiparallel forms. The influence of monovalent cations on G4 topology depends on the specific sequence, cation type, and concentration and structural features such as loop and flanking regions. For instance, Fujii et al. examined the influence of metal ions and crowding molecules on G-quadruplex topology.^46^ Their thermodynamic analyses indicated that Na^+^ stabilizes the antiparallel G4s, whereas K^+^ destabilizes this topology. The presented data suggest that metal cations regulate G-quadruplex topologies depending on the size of the metal-ion cavity and the degree of hydration.^46^ Based on *ab initio* analysis, it has been established that the coordination of metal cations contributes more to the stabilization of the G-quadruplexes than hydrogen bonds or G-tetrad stacking interactions.^51^ Early studies suggested that the preference for G4s formation decreases in the series K^+^ > NH_4_^+^ > Na^+^ > Li^+^ and K^+^ > Rb^+^ > Cs^+^.^52^

Beyond monovalent cations, divalent ions can also affect the formation of G-quadruplexes. For example, Sr^2+^ cations were found to promote dimerization in a four-stranded RNA G4 composed of UGGGGU strands.^53^ In turn, the structure of a four-stranded DNA G4 with the sequence d(BrU)r(GAGGU), containing a 5BrU modification, is stabilized by both Ba^2+^ and Na^+^ cations.^53^ Studies of the DNA G-quadruplex with the sequence d(G4T4G4) showed that in solution containing divalent cations, destabilization occurs in the order Zn^2+^ > Co^2+^ > Mn^2+^ > Mg^2+^ > Ca^2+^.^54^ On the other hand, stable RNA G4s formed in the presence of potassium ions were destabilized by increasing the concentration of divalent cations. The following order of destabilization strength was observed: Zn^2+^ > Cd^2+^ > Ni^2+^ > Co^2+^ >> Mn^2+^ > Mg^2+^ > Ca^2+^ > Sr^2+^ > Ba^2+^.^20^

Beyond the well-established roles of mono- and divalent cations in G-quadruplex stabilization, the redox reversibility of copper ions was recently suggested to act as a switch to modulate the G4 formation.^55^ Sahu et al. showed that Cu^+^ can induce guanosine to form a highly stable G-quartet/G-quadruplex structure that is distinct from the conventional K^+^-stabilized G4.^55^ They found that Cu^+^-induced G4 forms thermally stable supramolecular polymers in water. These polymers are stabilized by strong Cu^+^ coordination, Cu^+^–Cu^+^ metallophilic interactions, π–π stacking, and hydrophobic forces. Importantly, the formation and disassembly of the G4 structure can be reversibly regulated by redox switching between Cu^+^ and Cu^2+^.^55^ This finding may provide a useful basis for investigating redox-mediated regulation of G4 structures in biological systems.

## The effect of chemical modifications on G-quadruplexes formation

Chemical modifications of the heterocyclic base, sugar moiety, or phosphate backbone allow us to study the influence of each of these elements on the formation and stability of G4s. The effects of various types of modifications on G-quadruplex folding and stability are summarized in Table 1. We can distinguish three groups of modifications of guanosine residues.^56^ The first one disrupts or prevents the formation of a hydrogen bond between the O6 oxygen atom carbonyl group and the N1 hydrogen atom of two adjacent residues. This group includes 6-thioguanosine or O6-methylguanosine.^57^ The second group of modifications consists of nucleotide analogues such as inosine or 7-deazaguanosine, which prevent the formation of hydrogen bonds between N7 atoms and the NH_2_ group. The third group contains nucleotide analogues such as 8-oxoguanosine, 8-aminoguanosine, 8-bromoguanosine, or 8-methylguanosine.^58^ Residues with large substituents at the C8 position preferentially adopt a *syn* conformation; therefore, introducing them into the residues with the same orientation stabilizes the G4 structure. Additionally, it can also limit structural polymorphism by stabilizing the main conformer. The modifications of the sugar moiety include analogues such as LNA (locked nucleic acid), UNA (unlocked nucleic acid), 2ʹ-OMe-G (2ʹ-O-methylguanosine), 2ʹ-F-G (2ʹ-deoxy-2ʹ-fluoro-guanosine), or 2ʹ-FANA-G (2ʹ-deoxy-2ʹ-fluoro-arabinoguanine).^59^^,^^60^

**Table 1:** Summary of the effects of various chemical and structural modifications on G-quadruplex folding and stability

Part modified	Type of modification	Examples	General effect on stability	Reference
Heterocyclic base (guanosine)	disrupts O6-N1 hydrogen bonding	6-thioguanosine, O6-methylguanosine	destabilizing	Gros et al.^57^
	disrupts N7-NH_2_ hydrogen bonding	inosine, 7-deazaguanosine	destabilizing	Sagi^58^
	C8 position substituents	8-oxoguanosine, 8-aminoguanosine, 8-bromoguanosine, 8-methylguanosine	stabilizing (promotes *syn* conformation)	Sagi^58^
Sugar moiety	constrained sugar pucker (C3′-endo)	LNA (locked nucleic acid)	highly stabilizing, especially in G-tetrads	Roxo et al.^59^; Li et al.^60^; Randazzo et al.^61^
	flexible sugar pucker	UNA (unlocked nucleic acid)	destabilizing in G-tetrads; stabilizing in loops	Roxo et al.^59^; Li et al.^60^; Agarwal et al.^64^
	2′-position modification	2′-OMe-G (2′-O-methylguanosine)	generally destabilizing, especially for *syn-dG*	Ke et al.^63^; Zhao et al.^62^
		2′-F-G (2′-deoxy-2′-fluoro-guanosine)	context-dependent; often slightly destabilizing	Li et al.^60^; Assi et al.^202^
		2′F-ANA-G (2′-deoxy-2′-fluoro-arabinoguanine)	generally stabilizing	Li et al.^60^; Peng and Damha^203^
Phosphate backbone	replacement of the oxygen atom	phosphorothioates (sulfur substitution), methyl phosphonates	generally destabilizing	Novikova et al.^66^; Saccà et al.^65^
	linkage inversion	L-DNA monomers, 2′-5′ linkages, 3′-3' or 5′-5′ linkages	destabilizing	Novikova et al.^66^; Chilton et al.^67^; Su et al.^68^
Others	abasic sites	1′,2′-dideoxyribose, tetrahydrofuranyl residue	position-dependent; destabilizing; destabilizing	Virgilio et al.^204^; Školáková et al.^205^
	aliphatic linkers	propanediol, hexaethylene glycol groups	position- and length-dependent; stabilizing – intramolecular DNA G4, destabilizing – dimeric DNA G4; destabilizing	Risitano and Fox^38^; Cevec and Plavec^206^; Cevec and Plavec^206^

Overall, in G-quadruplexes containing the LNA residue, a significant stabilization was observed, caused by forced C3ʹ-*endo* sugar pucker conformation. However, this effect strongly depends on the location and number of such residues.^61^ It was reported that the introduction of LNA-T within a loop results more often in a slight enhancement of the G-quadruplex thermal stability when the modification is located near the G-tetrad.^59^ However, the most significant increase in G4 thermal stability occurred when the LNA was introduced directly within the G-tetrad.^59^ In turn, the presence of the 2ʹ-OMe group has a destabilizing effect on the G-quadruplexes.^62^^,^^63^ In 2014, Zhao et al. investigated how 2′-O-methyl nucleotides influence the folding topology and structural stability of the thrombin-binding aptamer (TBA) G-quadruplex under different environmental conditions.^62^ They found that single substitutions at *syn*-dG residues destabilized the G4 structure, whereas substitutions at *anti*-dG residues preserved the G-quadruplex formation in the presence of K^+^. Moreover, the destabilization effect of the 2ʹ-OMe group depended on its position within G4. The modification of one or two G-tetrads with 2′-O-methyl nucleotide led to an unstructured TBA.^62^ More importantly, the glycosyl torsion angle and the 2ʹ-OMe sugar pucker were found to be the most critical factors contributing to G4 structure destabilization.^63^

Other studies have shown that a single UNA modification in the G-tetrad markedly destabilizes the G4 structure, indicated by a significant decrease in melting temperature.^59^ Nevertheless, Agarwal et al. revealed that the effect of single UNA substitutions on G4s is position-dependent.^64^ They reported that UNA modifications within loops enhanced the global structure stability, whereas the UNA bases in the stem led to the significant G4 destabilization.^64^ This suggests that the UNA residues may be efficient modulators of the G-quadruplex thermodynamic stability.

The most common modification of the phosphate backbone is the replacement of the negatively charged oxygen atom in the phosphate group with another atom or functional group. In general, these modifications destabilize the G-quadruplex. The oxygen atoms of the phosphate backbone contribute to the formation of bridges between water molecules, the sugar moiety, and guanosine residues.^65^ Hydrogen bonding promotes an ordered distribution of water molecules along the grooves of the G4, which is important for its stabilization. The natural phosphate backbone can be modified in various ways, including the use of L-DNA monomers, 2′-5′-linkages, 3′-3′-linkage, 5′-5′-linkage methyl phosphonate linkages, methyl phosphoramidate, and replacement of non-bridged oxygen atom(s) with sulfur.^66^^,^^67^^,^^68^ It is known that not only the charge but also the ionic radius of the atom in the oligonucleotide backbone can affect G4 folding and stabilization. The modification of the phosphate backbone by substituting oxygen atoms with sulfur is suggested to influence G-quadruplex stability in a molecularity-dependent manner. Notably, the larger size of the sulfur in comparison with the oxygen in the bi- and tetramolecular phosphorothioate analogs destabilizes the G4 structure.^65^

## The role of the G4-specific ligands

Over the past few years, numerous small-molecule compounds have been designed for selectively binding G-quadruplexes, collectively referred to as G4 ligands. These compounds are typically characterized by a planar aromatic or heteroaromatic core, often conjugated with groups containing amino residues, quaternary ammonium groups, or pyridinium moieties. These structural modifications confer a cationic character to G4 ligands, improving their water solubility and electrostatic interactions with negatively charged nucleic acids.^69^^,^^70^^,^^71^

Depending on their structural organization, G4 ligands can be classified into three main families: (1) fused heteroaromatic polycyclic systems, (2) macrocycles, and (3) modular aromatic compounds.^71^ A majority of these ligands, along with detailed structural and biochemical data, are cataloged in the G4 Ligand Database (http://www.g4ldb.com/).

**Figure 2:**
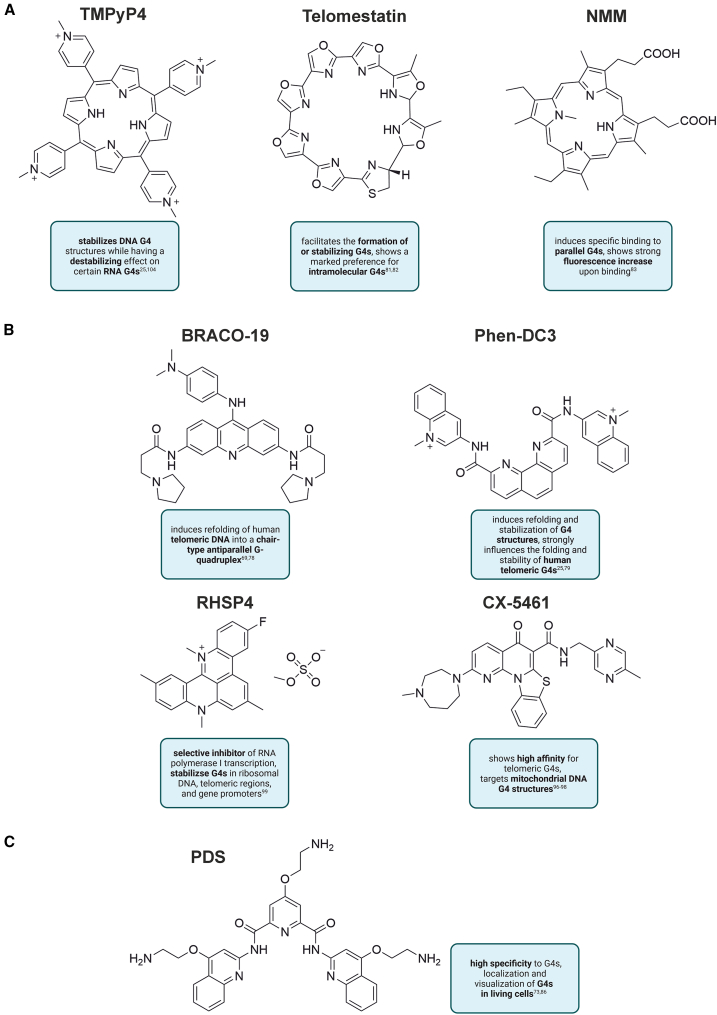
Chemical structures and selected properties of some known G4-specific ligands The compounds are grouped into three major structural families: (A) fused heteroaromatic polycyclic systems, (B) macrocycles, and (C) modular aromatic compounds.

Typically, binding of a G4 ligand to the G-quadruplex enhances its stability, although this effect depends on the ligand properties and the G4 structure. Overall, understanding how different ligands interact with various G4 topologies remains crucial for improving selectivity and therapeutic potential. Some examples of well-characterized G4-specific ligands are shown in Figure 2.

Many well-known ligands, such as TMPyP4 [5,10,15,20-tetrakis-(N-methyl-4-pyridyl)porphine; Figure 2A] or Phen-DC3 (bisquinolinium compound; Figure 2B), rely on π−π stacking interactions between their aromatic core and G-tetrads.^34^^,^^72^^,^^73^ It is now clear that other interactions, including groove, loop, or cation-π interactions, can also contribute substantially to G4-ligand binding.^74^^,^^75^ For instance, Liu et al. demonstrated NMR solution structures of pyridostatin (PDS; Figure 2C) and its derivatives (PyPDS) in complex with G-quadruplex-duplex hybrid.^76^ Structural analysis revealed that the rigid aromatic rings of PyPDS/PDS, linked by flexible amides, stack adaptively on G-tetrads, enhancing π–π interactions and specific G4 recognition. Their aliphatic amine side chains adopt conformations stabilized by electrostatic and hydrogen-bond interactions with the phosphate backbone, increasing their affinity for G4s.^76^ Another example of a G4/ligand complex was reported by Lin et al., who performed biophysical and X-ray structural studies of the DNA G-quadruplex in complex with the N-methylmesoporphyrin IX (NMM) compound (Figure 2A).^77^ The authors investigated the interaction of a G-rich sequence with NMM and solved the crystal structures of complexes, supporting the 1:1 binding stoichiometry.^77^

Some ligands exhibit selective binding and functional effects on G-quadruplex structures. TMPyP4, for example, stabilizes RNA G4 structures in the hepatitis C virus genome, resulting in dose-dependent inhibition of viral RNA levels.^78^ However, it has been previously reported that TMPyP4 can interact with duplex DNA and other non-G4 structures.^79^ Moreover, several studies reported that this ligand exhibits only a modest, a few-fold preference for G-quadruplex over alternative DNA structures, which can explain its limited selectivity in cellular applications.^80^ Phen-DC3, in turn, induces topological transitions in human telomeric DNA, refolding hybrid G4s into antiparallel chair-type structures.^81^ BRACO-19 (Figure 2B) preferentially stacks onto telomeric DNA G4s, promoting dimerization and telomerase inhibition.^82^ The natural macrocycle telomestatin (Figure 2A) demonstrates high selectivity toward G4 DNA over duplex DNA.^83^^,^^84^ Rosu et al. studied telomestatin binding to DNA G4 and observed that an extra ammonium ion is incorporated into the G-quadruplex structure.^85^ Furthermore, the NMM ligand possesses two propionic acid side chains, which are likely negatively charged at physiological pH. This compound selectively binds parallel G4s, a process often monitored via fluorescence enhancement.^86^

Recent developments have significantly broadened the landscape of G4-specific ligands.^45^^,^^87^^,^^88^ Among these, compounds such as carboxy-pyridostatin,^89^ QUMA-1,^90^^,^^91^ RGB-1,^92^ and naphtho-TASQ^93^ have been shown to specifically recognize parallel-stranded RNA G4s. These compounds combine planar aromatic cores for π–π stacking with G-tetrads, along with flexible side chains or functional groups that facilitate additional hydrogen bonding or electrostatic interactions with the loops and grooves of the RNA G4.^87^^,^^89^^,^^90^^,^^92^^,^^93^ This structural complementarity not only stabilizes the RNA G4 fold but also enhances selectivity over DNA G4s and other non-G4 nucleic acid structures. Such high specificity is essential for investigating the biological functions of RNA G-quadruplexes in processes such as translational regulation and mRNA localization, as well as for the prospective development of RNA-targeted therapeutics.

Although early ligands such as TMPyP4 and pyridostatin showed strong selectivity for G4 DNA over dsDNA, the main challenge today is achieving “intra-G4 selectivity”, meaning the ability to distinguish between different G4 structures in the genome. Several examples from the literature show progress in this direction. Some ligands (such as GQC-05 and CD-34) preferentially stabilize the G4 structure in the promoter of the c-MYC oncogene rather than other promoters or telomeric G4s.^94^^,^^95^^,^^96^ Other molecules are designed to distinguish between the Pu27 G-quadruplex from the c-MYC promoter and the telomeric Telo21 G-quadruplex.^97^^,^^98^ This selectivity arises from interactions with loop or flanking regions rather than only the G-tetrad core. In addition, some ligands show preferences for particular G4 topologies, for example, macrocyclic systems and V-shaped squaraine derivatives that favor parallel over antiparallel folds.^99^^,^^100^ Metal-based complexes can selectively stabilize higher-order telomeric G4 assemblies, while natural products such as sanguinarine recognize G4s in a topology-dependent manner.^101^^,^^102^

Further supporting this trend, Das and Chorell reported a comprehensive analysis of pyridine bis-quinazoline derivatives as selective G-quadruplex DNA stabilizers.^103^ The findings showed a strong G4 stabilization and selectivity over dsDNA upon the novel bis-quinazoline derivatives in a dose-dependent manner. Moreover, these results underline the important role of the presence and composition of the aliphatic amine side chain in achieving effective G-quadruplex stabilization.^103^

Interestingly, a distinct class of ligands, known as photoswitchable G4 ligands, has been developed to provide dynamic, light-controlled modulation of G4 structures.^104^^,^^105^^,^^106^ These compounds contain photoresponsive moieties, typically azobenzene or related chromophores, that undergo reversible conformational changes upon irradiation with specific wavelengths of light. This conformational switch alters the ligand binding affinity or geometry, enabling temporal and spatial control over G4 stabilization.^106^ One example of such a ligand is a photoresponsive dithienylethene (DTE).^107^ Galan and co-workers reported the first G4-specific enhancement of photoswitching kinetics. They also demonstrated that the binding mode can be switched using visible light, enabling the regulation of telomeric G4 tetrad structure in K^+^ buffer. They found that the process is fully reversible, thereby eliminating the requirement for high-energy UV light.^107^ Unlike conventional static ligands, photoswitchable G4 ligands enable researchers to precisely regulate G4 folding, ligand-G4 interaction kinetics, and downstream biological processes in living cells or *in vitro* experiments. This approach represents a significant advance in the G4-specific ligand landscape. It enables light-controlled modulation of nucleic acid structures and precise regulation of gene expression, replication, or telomere function.

Interesting examples and recent advances in G-quadruplex ligand design include CX-5461 [2-(4-methyl-[1,4]diazepan-1-yl)-5-oxo-5*H*-7-thia-1,11*b*-diaza-benzo[*c*]fluorene-6-carboxylic acid (5-methyl-pyrazin-2-ylmethyl)-amide, Pidnarulex; Figure 2B] and RHPS4 (3,11-difluoro-6,8,13-trimethyl-8H-quino[4,3,2-kl]acridinium methosulfate) (Figure 2B), which highlight the therapeutic potential of G4 targeting.^108^ CX-5461 is a planar aromatic G-quadruplex ligand that was originally identified as a selective inhibitor of RNA-polymerase-I-dependent transcription.^109^ Interestingly, this compound is the first G4-stabilizing molecule to enter clinical trials.^110^ It stabilizes G-quadruplex structures via π–π stacking terminal G-tetrads, resulting in replication stress and activation of DNA damage responses in cancer cells.^110^^,^^111^ In contrast, RHPS4, a cationic polycyclic acridine derivative, is distinguished by its mitochondria-specific accumulation.^112^^,^^113^ By selectively stabilizing mitochondrial DNA G-quadruplexes, RHPS4 inhibits mitochondrial replication and transcription, resulting in mitochondrial dysfunction and cell death.^112^^,^^113^^,^^114^ This compound targets both telomeric and telomerase-associated G4s, inducing telomere dysfunction.^112^^,^^114^ Together, these ligands illustrate recent advances in ligand design that enable both clinical translation and subcellular specificity in G-quadruplex targeting.

To conclude, this section summarizes the key factors influencing the formation and stabilization of G-quadruplexes. It also explains the impact of chemical modifications on G4 structures and briefly explores the role of ligands that can bind both DNA and RNA G-quadruplexes. The growing number of such ligands and recent advances in their development underline their potential in many therapeutic applications. However, many G4-binding ligands exhibit limited selectivity. As a result, they may bind multiple G-quadruplexes or other nucleic acid structures, thereby restricting precise targeting in cellular environments. Therefore, alternative strategies that provide higher sequence specificity are being explored. Among these, ASO-based approaches represent a promising direction for selectively targeting and regulating G4s. Thus, the next section focuses on the ASO-based strategy for G-quadruplex targeting.

## Antisense oligonucleotides as tools to modulate G-quadruplex structures and their therapeutic potential

ASOs are short DNA or RNA strands that, due to their sequence-specific complementarity, hybridize to target nucleic acid sequences, thereby altering biological processes. Notably, ASOs exhibit high specificity for their target sequences, binding to them via Watson-Crick base pairing.^115^ In cells, ASOs inhibit gene expression through different mechanisms of action. These oligonucleotides can either recruit RNase H to induce RNA cleavage, trigger RNA interference pathways, or act as a steric blockage preventing the mRNA-ribosome interaction.^116^ To date, given their unique features, ASOs have been widely used in the treatment of many diseases. One important group of disorders for which the ASO-based therapy was developed includes neurodegenerative diseases, e.g., motor neuron diseases like spinal muscular atrophy (SMA) or amyotrophic lateral sclerosis (ALS).^117^ The ASO-based drug—nusinersen, which acts as a splicing modulator, has already been approved by the Food and Drug Administration (FDA) for treatment of SMA, a disease caused by exon skipping and, consequently, low protein levels.^117^

Due to the importance of G-quadruplexes in cellular processes and their highly dynamic nature, these structures have gained interest as targets in ASO-based therapies. Additionally, a major benefit of using the antisense approach to target G4s, rather than, for example, G4-specific ligands, is the reduction of potential off-target effects within the cell. This reflects the observation that numerous G4 structures form *in vivo*, and most ligands recognize the G4 topology rather than specific sequences. In contrast, ASOs are designed to selectively bind to a G-quadruplex-forming sequence of interest, allowing a specificity toward the target.^118^

**Figure 3:**
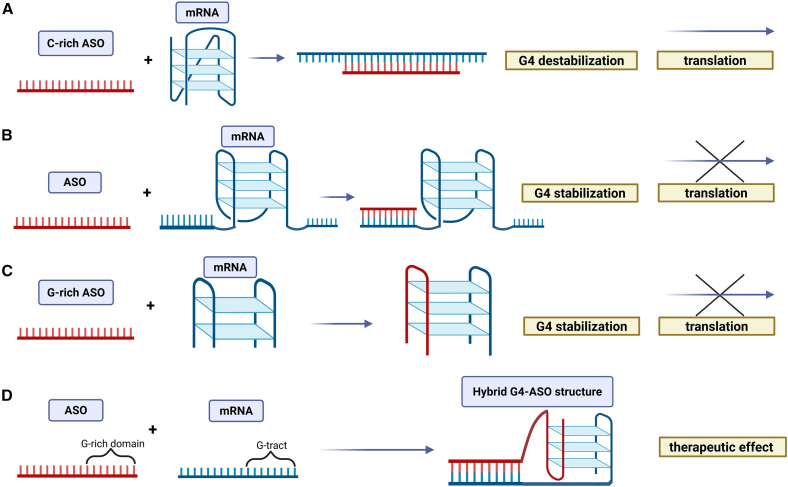
A schematic representation of ASO-based strategies to modulate RNA G4 formation and affect the translation of specific mRNAs (A) ASO binding to mRNA prevents G4 formation, enabling translation to proceed, (B) ASO binding adjacent to a G4 motif promotes its stabilization, acting as a steric barrier to translation, (C) G-rich ASOs target pre-formed G4 structures and further stabilize them, (D) ASO binding induces *de novo* G4 formation in regions that do not naturally adopt this structure, leading to translational repression.

Several strategies have been developed to modulate G-quadruplex structures in cells. The choice of approach depends on whether the G4 formation, stabilization, or disruption is desired. As shown in Figure 3, various ASO-based strategies can be employed to modulate G-quadruplex structures. Multiple factors, including the location of the target G4-forming sequence, the underlying molecular mechanism of the disease, and the functional role of the G4 structure in the biological process, influence the choice of ASO-based strategy.

The first approach involves ASO binding to the mRNA sequence to prevent its folding into a G-quadruplex structure, allowing translation to proceed (Figure 3A). Conversely, ASO binding to the sequence adjacent to the G4 structure can promote motif stabilization, acting as a steric block during translation (Figure 3B). Some ASOs are designed to target pre-formed structures to suppress translation efficiency. The use of G-rich ASO can stabilize the preexisting G4 structure, for instance, by an additional G-tetrad formation (Figure 3C). In some cases, the ASO binding can induce G-quadruplex formation in regions that do not normally adopt this structure in the biological environment, which may cause the expected therapeutic effect (Figure 3D).^118^^,^^119^

The first ASO-based strategy targets G-quadruplexes in the cell to prevent their formation.^120^ It can employ cytosine-rich oligomers or their analogs that bind to G-tracts in sequence. This approach can lead to G4 destabilization and, consequently, to increased protein expression.^120^ Similarly, Chowdhury et al. designed LNA-modified ASOs that efficiently destabilized G4s, leading to the increased c-KIT protein expression in cellular models.^121^ Importantly, the addition of LNA strongly promoted and accelerated the disruption of the G4 structure.^121^ Beyond its impact on translation efficiency, inhibiting G-quadruplex formation can also cause decreased transcription levels. It has previously been shown that delivery of ASO complementary to PQS motifs into zebrafish embryos results in binding to a G4-forming sequence located within the promoter region.^122^ This, in turn, disrupts the naturally occurring G-quadruplex structure in this region, ultimately resulting in decreased transcription.^122^

In addition to ASO binding to sequences adjacent to G-quadruplex structures, another potential strategy for modulating G4s involves designing G-rich ASOs. These ASOs enhance G4 stability by reprogramming the existing G-quadruplex conformation.^123^ ASOs designed this way contain guanine stretches on one end that are not complementary to the target sequence.^123^ However, these guanines recruit G residues from the target mRNA sequence to finally form the G-quadruplex structure. Thus, for instance, binding of such G-rich ASOs can cause the formation of an additional G-tetrad in the structure. These more stable G4s can significantly enhance translation inhibition in cells, thereby reducing protein levels.^123^

A similar strategy can be employed to promote DNA-RNA heteroquadruplex formation within the regions that typically do not adopt a G4 structure *in vivo*. This strategy uses a DNA oligonucleotide composed of two domains. One domain is an antisense region that hybridizes to the target RNA, and the other is a G-rich segment that forms a G4 structure with the G-tract in RNA.^124^ Such a heteroquadruplex may cause structural blockage in biological processes or a change of RNA function.^119^ In contrast, traditional ASOs primarily act via Watson-Crick base-pair complementarity with the target RNA. Therefore, conventional ASOs are not capable of promoting the formation of the G4 structure but may instead stabilize or disrupt a preexisting G4 motif.^125^

An approach that induces heteroquadruplex formation can be used to block RNA reverse transcription, for example, during HIV-1 replication.^120^ A similar approach was utilized to inhibit translation of the EGFR gene.^119^ The addition of chemically modified ASO to the mRNA molecule containing G-tracts caused the formation of a G-quadruplex-duplex hybrid. The G4 structure formed in this manner was confirmed to act as a steric block during translation.^119^

Additionally, some ASO-based splicing modulators act by promoting G-quadruplex formation in the cell. In this case, the G-quadruplex-inducing ASOs, consisting of two domains, one complementary to the target mRNA and the other forming a G4 motif with a G-tract present within the sequence, cause the inhibition of alternative splicing by blocking access to splicing factors.^126^ Therefore, they can be applied in gene therapies, for example, in the treatment of Duchenne muscular dystrophy.^126^

Specific mRNA regions are often targeted preferentially in ASO-based therapeutic strategies. The 5′-UTR of mRNA is the site of ribosome binding during translation initiation; therefore, any changes in this region can directly affect protein levels.^127^ Many mRNAs contain G-rich sequences in their 5′-UTRs that can fold into RNA G4 structures. Therefore, these motifs are known structural elements influencing translation. Interestingly, G-quadruplexes within 5′-UTRs usually act by blocking ribosome scanning or interfering with the assembly of the initiation complex.^127^ Extensive research demonstrates that targeting G4s formed within the 5′-UTR by ASO can stabilize these structures and, in turn, affect protein expression levels, typically leading to downregulation.^128^

Turcotte and Perrault designed a G-rich ASO that interacts with and reprograms an RNA G4 in the 5′-UTR of the monoamine oxidase B mRNA.^123^ The binding of the ASO converts the native two-quartet G-quadruplex into a more stable three-quartet one. This structural stabilization enhances translational repression mediated by the G4 compared with the unstabilized G-quadruplex.^123^ In a similar context, ASOs can also be used as tools to reveal the correlation between the RNA G-quadruplexes formation and translational repression. In such an experiment, ASO is designed to prevent the formation or unfold the G4 structure present within the 5′-UTR region. Consequently, the disruption of naturally occurring G-quadruplexes restored translation.^129^

Remarkably, the specificity of ASOs toward G-quadruplexes can have alternative therapeutic applications. For instance, G4-forming sequences can be introduced into the micelles, which are potential drug carriers in cells, to stabilize them.^130^ These modified particles can be selectively disrupted by ASOs, releasing the cargo at the site of interest.^131^ Besides using ASO targeting G-quadruplexes as therapeutic agents, they may also be utilized to identify G4s and assess their localization in cells.^132^ In this approach, ASOs bind to G4s and unfold the structure. By targeting these sequences, ASOs disrupt G4 structures, which would otherwise serve as targets for fluorescent probes. The decrease in fluorescence signal confirms the presence of a G-quadruplex in a specific genome region.^132^

Notably, most of the ASO-based strategies act at the RNA level. However, ASO can also interact with G-quadruplexes in DNA, modulating transcription. Using this approach, the transcription of certain genes, in which G4 structures play a crucial role in transcription regulation, can be reduced, including oncogene transcription.^133^ The underlying mechanism involves the preferential formation of a DNA duplex upon ASO binding, thereby disrupting G-quadruplex folding.^134^

Due to the high genetic variability of viral genomes, there is a constant need to identify new targets for antiviral drugs. In addition to targeting viral proteins, recent research has significantly focused on structural elements within viral genomes that play critical roles in the viral life cycle.^135^ Furthermore, therapeutic agents targeting these structural motifs should be highly selective, minimizing off-target effects in infected cells. Viral genomes have been shown to adopt multiple secondary and tertiary RNA/DNA structures.^136^^,^^137^^,^^138^ Viruses with RNA genomes are particularly known for their large structural and functional diversity.^139^^,^^140^ As previously described, G-quadruplex structures are present within viral genomes and appear to play significant roles in the viral replication cycle. Therefore, they represent promising novel targets for antiviral drug development, including potential therapeutic strategies against SARS-CoV-2 infection.^140^^,^^141^ Notably, ASOs have been widely used in antiviral research due to their versatility and rapid adaptability to emerging viruses and diverse viral variants.^142^ These molecules can be designed to target various regions within viral genomes, including the potential quadruplex-forming sequences. Furthermore, ASOs can modulate host cellular factors that are essential for viral propagation. This flexibility allows ASOs to be designed to act at various stages of the viral life cycle.^142^ In viruses, as in the other research subjects, ASOs can target G-quadruplexes within mRNA, thereby affecting viral protein translation. In the case of Epstein-Barr virus, ASO binding destabilizes G4 structures within mRNA, leading to increased Epstein-Barr-virus-encoded nuclear antigen 1 synthesis and antigen translation.^143^ This approach represents a novel immune-activating strategy by enhancing antigen translation. As a result, antigen presentation is enhanced, thereby enabling more efficient recognition of viral infections by the host immune system.^143^ Using a similar strategy, the G4 structure within the latency-associated nuclear antigen (LANA) mRNA of Kaposi-sarcoma-associated herpesvirus was disrupted after ASO binding.^144^ This resulted in increased protein expression.^144^

Alternatively, an ASO targeting viral genetic material can promote G-quadruplex formation, thereby reducing viral infectivity. As described above, such oligonucleotides contain the antisense domain and the guanine stretch that, along with viral RNA, form a G-tetrad. The formation of RNA-DNA heteroquadruplex promoted by G-rich ASO addition within the HIV-1 inhibits a critical step of viral replication: reverse transcription.^145^ An example of the G-quadruplex present within the cellular host factor that can be important for viral infection is the G4 structure formed within the TMPRSS2 protein, which has serine protease activity essential for SARS-CoV-2 infection.^146^ ASO directed toward this G-quadruplex could unwind this structure.^146^

However, despite their beneficial properties, the use of ASOs in cellular environments has certain limitations. Intracellular delivery of ASO is a major limitation that should be addressed when considering targeting of G-quadruplexes in cells. ASOs are relatively large, negatively charged molecules with limited permeability across the cell membrane.^147^ Therefore, they cannot cross plasma membranes without any additional carriers, such as lipofectamine, and, more importantly, they can be easily degraded by enzymes present in the cell. To overcome these issues, various chemically modified residues are incorporated during the ASO design process. Among the most popular modifications are peptide nucleic acid (PNA), LNA, phosphorodiamidate morpholino (PMO), and 2′-O-methyl/2′-O-methoxyethyl moieties.^148^ Other approaches involve incorporating structural modifications into ASO in the form of a G-quadruplex motif.^4^ In this design, the ASO retains the Watson-Crick base pairing region, while the appended G4 structure increases molecular stability and binding affinity to the target mRNA. Moreover, the linkage between the G4 motif and the ASO enables the controlled release at an intracellular level. This structurally optimized ASO can be used as a therapeutic agent in anticancer applications.^4^ Another study showed that a G-rich ASO adopts the G4 structure, which was developed to bind and inhibit the function of nucleolin, a protein overexpressed in many cancer cells.^149^

Another limitation to consider when targeting G-quadruplexes is their high structural variability. In some cases, the same target sequence can adopt various G4 folding topologies.^150^ As a consequence, ASO is designed solely based on sequence complementarity, or recognizing only one structural state may modulate G-quadruplex structures under specific conditions.^148^ An additional challenge in targeting pre-formed G-quadruplexes with ASO is the restricted accessibility of nucleotides that are complementary to the ASO within the folded structure. In stable G4, guanine residues involved in tetrad formation are located within the G-quadruplex core. Thus, they are unavailable for Watson-Crick base pairing, which reduces the efficiency of ASO hybridization.^151^^,^^152^

Targeting G4 structures with ASO is often further complicated by competition with endogenous RNA-binding proteins (RBPs) that recognize and bind G-quadruplexes in cells. The binding of these proteins can block access of ASO to complementary nucleotides. In addition, RBPs bind to G4s with high affinity, potentially reducing the efficacy of exogenously delivered ASO.^153^ Moreover, the complexity of the cellular environment, where many G-quadruplex motifs and other RNA structures coexist, significantly influences ASO binding, increasing the risk of off-target effects.^118^ Rouleau et al. assumed that even short ASOs (∼15 nucleotides) can bind the mRNA G4-forming region, affecting the folding of the G-quadruplex structure.^118^ At the same time, the usage of shorter ASO can limit the off-target effects.^118^ They selected several target sequences, in some instances within the 5′-UTR regions. The goal was to establish the impact of such ASO on mRNA translation in cells. The authors successfully demonstrated that using shorter ASO targeting G4s allows for promoting or stopping translation depending on the target.^118^

Overall, despite many *in vitro* studies, the efficacy of ASO targeting G-quadruplexes remains poorly validated *in vivo*. Factors such as ASO cellular uptake and stability under cellular conditions, G4 interactions with proteins, and formation of alternative structures lead to variable pharmacokinetics and pharmacodynamics. This, in turn, limits the translation of *in vitro* effects to animal models and clinical trials.^154^

In summary, ASOs can stabilize or disrupt G4s, form hybrid G4 structures, or be combined with ligands to improve the interaction specificity. These features are being exploited for gene regulation, antiviral strategies targeting viral G4s, and the development of therapeutics. Together, ASO-based strategies demonstrate significant therapeutic potential for modulating G-quadruplex structures in both cellular and viral contexts. However, it must be taken into account that a folded G-quadruplex structure may be inaccessible to ASO, which is designed only based on the sequence complementarity. For this reason, additional complementary strategies should also be explored to overcome this limitation. Therefore, in the next chapter, we discuss ligand-ASO conjugates as a promising dual-function strategy. This approach combines the sequence specificity of ASOs with the structural recognition of G-quadruplex-binding ligands, enabling more precise targeting and modulation of G-quadruplex structures.

## Ligand-ASO conjugates as dual function strategies for precise G-quadruplex targeting and modulation

As described in previous chapters, ASOs represent a promising and versatile strategy for the targeted modulation of G4 structures. In the following section, we will explore the rationale, design strategies, and recent progress in the development of ligand-ASO conjugates as a next-generation platform for G4 targeting, with the emphasis on their biochemical properties, molecular mechanism of action, and potential biomedical implications.

**Figure 4:**
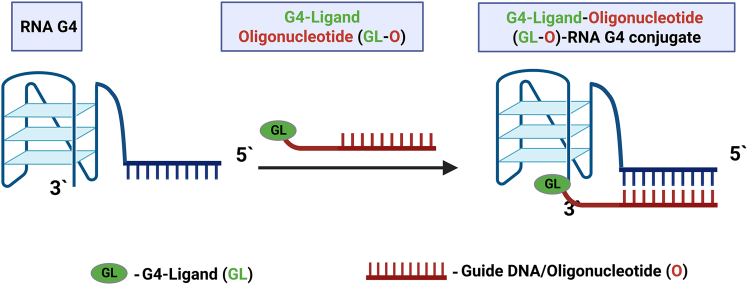
A general representation of the strategy based on targeting individual RNA G4 structures using G4-ligand-oligonucleotide (GL-O) conjugates

Unlike small-molecule G4-specific ligands, which often suffer from low selectivity and a high propensity for off-target effects, ASOs offer a sequence-specific mechanism of action. This property makes them particularly attractive for applications requiring precise recognition and modulation of G4-forming sequences. Despite several advantages of the ASO-based approach, such as high specificity and adaptability, challenges remain, including cellular delivery, target accessibility, and residual off-target binding. In this context, an innovative and more effective strategy is emerging—the development of ligand-ASO conjugates (Figure 4). These hybrid molecules combine the sequence-specific binding capability of ASOs with the structural recognition properties of G4-binding ligands.

The conjugation of ASOs with selective G4 ligands is designed to enhance overall binding affinity and specificity toward particular G4 structures, while simultaneously reducing undesired interactions with non-target regions. This dual-functionality approach has significant potential for improving the precision of G4 targeting. It may also expand the therapeutic applicability of antisense technologies in diseases where G-quadruplexes play a regulatory or pathogenic role.

Berner et al. developed a selective strategy for targeting individual G4 structures using G4-ligand-oligonucleotide conjugates (GL-Os).^155^ These conjugates combine a G4-binding ligand with a short oligonucleotide complementary to the sequence flanking the target G4. The dual binding, i.e., ligand to the G4 core and oligo to the adjacent sequence, ensures high specificity and enhanced stabilization. This modular approach allows discrimination among structurally similar G4s and enables precise targeting for potential applications in G4 biology and therapeutic development.^155^

In recent years, G4-based targeting has emerged as a promising approach for enhancing the selectivity and efficacy of ASO therapies in oncology. Recently, two complementary strategies of integrating G4 chemistry into ASO design were proposed to overcome challenges in cellular uptake, degradation, and structural selectivity.^4^^,^^156^ Chen et al. developed an ASO prodrug Mal_2_-G4-ASO, in which the ASO is conjugated with a maleimide-functionalized G4-ligand.^4^ This design allows the G4 structure to assemble at the prodrug’s termini, enhancing stability and lysosomal escape, facilitating cellular uptake, and improving therapeutic efficacy in oncological applications.^4^

Chorell and co-workers created G4-ligand-conjugated oligonucleotides (GL-Os) using various G4-binding ligands, such as NMM and thiazole orange.^156^ The ligands are linked to the oligonucleotide via flexible linkers, providing enhanced G4-binding specificity and high stability, even under thermal or structural stress, allowing precise targeting of oncogenic G4s.^156^ Both G4-mediated delivery strategies and direct G4-targeting approaches offer significant advantages for cancer treatment.

Previously, a strategy for targeted delivery of ASOs by conjugating them to small-molecule ligands such as anisamide, which specifically bind to sigma receptors overexpressed in tumor cells, has been proposed.^157^ The reported approach involved direct incorporation of the ligand into the oligonucleotide via solid-phase synthesis, allowing for efficient mono- and trivalent conjugate formation. The multivalent conjugates offer high precision, scalability, and low toxicity, making them an attractive tool for therapeutic applications and the selective modulation of gene expression.^157^

In 2025, Gołębiewska-Pikuła et al. described a phosphate-triester-based platform for late-stage, multifunctional oligonucleotide modification, enabling the incorporation of G4-binding ligands, fluorophores, biotin, and other chemical groups.^158^ This solution-phase method bypasses solid-phase synthesis, offering flexibility and compatibility with phosphorothioate backbones. Notably, the resulting G4-ligand-oligonucleotide conjugates retain G-quadruplex binding activity while gaining additional functionalities, making this approach particularly useful in chemical biology, imaging, and target-specific applications.^158^

In addition to silencing or targeting specific G4s, ASO-ligand conjugates have been developed to recover or stabilize defective G4 motifs. A notable example is the study by Takahashi et al., in which G-rich oligonucleotides were conjugated with the G4-specific ligand, Phen-DC3, to rescue the formation of mutated or destabilized G4 structures.^159^ These conjugates, referred to as ligand-conjugated guanine oligonucleotides (LCGOs), were designed to hybridize to complementary DNA or RNA sequences and simultaneously induce or stabilize parallel or hybrid G4 topologies, even in the presence of G→T mutations or loop alterations that disrupt native G4 folding. The study demonstrated that the covalent attachment of Phen-DC3 via a flexible linker (e.g., through a C3 spacer) significantly improved the thermal stability and formation efficiency of G4s, as confirmed by circular dichroism spectroscopy, thermal denaturation profiles, and gel mobility shift assays. This dual-function design combines the sequence-specific recognition of the ASO with the structural reinforcement provided by the ligand. This approach represents a unique strategy for restoring functional G-quadruplexes, which are important for gene regulation and in disease-associated genomic regions.^159^

G-quadruplexes not only regulate human gene expression but also play important roles in viral genomes. In viruses, G4s often control key stages of the life cycle, including replication and assembly.^160^ This makes viral G4s promising targets for broad-spectrum antiviral strategies. For example, Xie et al. identified a highly conserved G4 structure in the tobacco mosaic virus (TMV) genome that is essential for viral proliferation.^161^ Using a combination of biophysical assays and plant-based functional studies, the authors demonstrated that targeting this motif could effectively suppress viral replication. To achieve this, they designed a multi-functional conjugate combining a G4-ligand, ASO, and a photosensitizer. This agent precisely recognizes TMV G4 and, upon light activation, generates localized reactive oxygen species that damage the viral RNA. This dual-action approach, combining structural stabilization with light-triggered phototoxicity, highlights the potential of G4-targeted conjugates as a controllable and highly selective platform for antiviral therapy.^161^

In summary, ligand-oligonucleotide conjugate strategies represent an emerging and promising direction for G4-targeted applications. By combining the sequence specificity of ASOs with the structural recognition of G4-binding ligands, these hybrid molecules overcome the limitations of conventional small-molecule ligands and broaden the functional potential of ASOs. Recent studies highlighted the versatility of this approach, demonstrating improved stabilization, superior selectivity, and potential for multifunctional applications (e.g., photodynamic therapy).^156^^,^^162^ Despite its promise, clinical application of this field remains limited. To date, very few ligand-ASO constructs have been validated in physiologically relevant models, with most studies relying on *in vitro* assays or high-specificity plant models.^4^^,^^156^^,^^163^ Major challenges, including the metabolic stability of the conjugates, efficient delivery to target tissues in mammals, and the long-term safety of these hybrid structures, have yet to be fully addressed. Bridging the gap between these innovative proof-of-concept designs and complex animal models will be essential to utilize their potential as precise therapeutic interventions within disease-relevant genomic regions. Ultimately, if these translational challenges can be overcome, ligand-oligonucleotide conjugates will offer a powerful platform for the selective targeting of individual G4s, enabling both novel therapies and the functional study of specific G4s within complex genomic contexts.

## Peptide nucleic acids and their conjugates as potential therapeutic agents for G-quadruplex modulation

**Figure 5:**
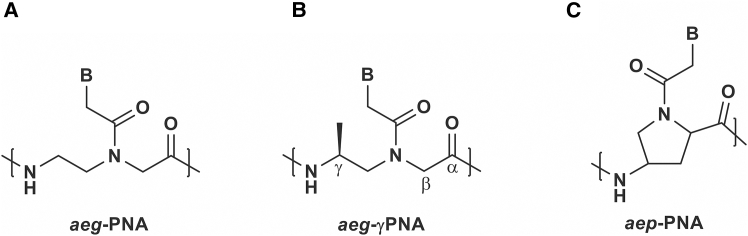
A chemical structure of peptide nucleic acid (PNA) and representative modifications (A) unmodified PNA (aeg-PNA), (B) gamma-modified PNA (aeg-γPNA), and (C) aminoethylprolyl PNA (aep-PNA); in all structures, letter B indicates a nitrogen base.

Another type of molecule evaluated as a tool for inhibiting G4 activity is peptide nucleic acid (PNA). PNAs (Figure 5A) are synthetic analogues of naturally occurring nucleic acids, RNA and DNA. From a structural perspective, these molecules combine features of both nucleic acids and proteins: they contain nitrogenous bases typical of nucleic acids, and their monomers are joined by amide bonds, as in proteins. Instead of negatively charged deoxyribose/ribose phosphate backbones, they include neutral N-(2-aminoethyl)glycine units.^164^ Due to their neutral backbone, they exhibit highly favorable hybridization properties with both RNA and DNA,^165^ making them promising molecules for an antisense approach. One of their limitations is a low rate of cell membrane penetration.^166^^,^^167^ Therefore, the improvement of cellular uptake is achieved by the introduction of positively charged amino acids (e.g., lysine), cell-penetrating peptide (CPP),^168^^,^^169^^,^^170^ or neamine to the molecule of PNA.^171^^,^^172^

Beyond G4 induction or strand invasion, short PNA sequences can be strategically designed to target the unique, long loop of G-quadruplexes, such as the five-residue loop-4 found in the human c-kit promoter.^173^ Research by Amato et al. demonstrates that the therapeutic mechanism of these short PNAs is critically dependent on both the sequence length and the nature of the stabilizing cation (K^+^ vs. NH_4_^+^).^173^ In a potassium-rich environment, where the G4 structure is highly stable, short PNAs (5- to 8-mers) can act as G-quadruplex-binding agents. These probes stabilize the pre-existing G4 scaffold by forming Watson-Crick base pairs with specific loop residues without disrupting the G-tetrad core.^173^ Conversely, under less stable conditions, such as NH_4_^+^-rich solution, the same short PNAs can act as “G-quadruplex openers.” They overcome G4 structure by forming PNA/DNA hybrids that, as PNA length and concentration increase, assemble into 2:1 triplex-type structures.^173^ This cation-dependent molecular switch suggests that short PNAs targeting long loops represent a refined approach for specific modulation of gene expression by either stabilizing or unfolding the G4 target based on the local thermodynamic stability.

Crucially, the ability of short PNAs to invade a G4 is also a kinetically controlled process. These molecules can successfully compete with G4 formation if present during annealing, but they are often unable to unfold a preformed G-quadruplex at room temperature.^174^ This highlights that the ionic environment, specifically the type and concentration of cations such as K^+^ and NH_4_^+^, serves as a “molecular switch” that determines the PNA therapeutic mechanism.

To increase the specificity of binding between nucleic acid and PNA, the basic structural framework of PNA has been chemically modified. The introduction of a chiral center at the γ position (Figure 5B) was found to induce a pre-organized right-handed helical conformation, thereby improving the hybridization properties of the molecule.^175^ Another interesting modification, aminoethylprolyl PNA (aep-PNA; Figure 5C), is a chiral and conformationally constrained analogue, known to form stable G-quadruplexes.^176^

**Figure 6:**
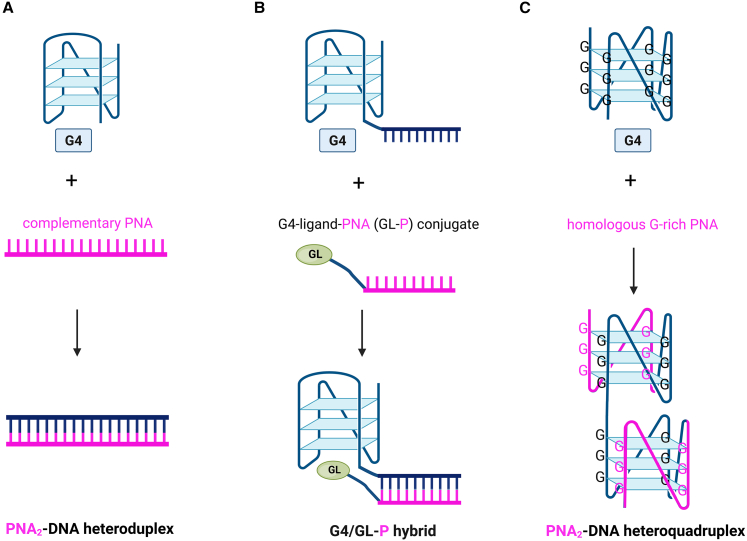
A schematic representation of the most explored PNA-based strategies affecting the G-quadruplex mode of action (A) the use of complementary PNA to form a heteroduplex, analogous to a conventional antisense oligonucleotide approach, (B) the incorporation of ligand–PNA conjugates to enhance the binding with the G4 target, and (C) the use of homologous PNA sequences to promote heteroquadruplex formation.

In general, several approaches have been proposed that utilize PNAs to interfere with G-quadruplexes. Figure 6 illustrates three main strategies: (A) the use of a complementary PNA to form a heteroduplex (analogous to a well-established ASO approach), (B) the use of ligand-PNA conjugates (an improvement of the first strategy), and (C) the use of a homologous PNA to form heteroquadruplexes. The first two strategies have been explored most extensively and therefore constitute the primary focus of this review.

The first approach (Figure 6A) is based on Watson-Crick base-pairs between two complementary strands of nucleic acids. Cytosine-rich PNA interacts with G-quadruplexes via hydrogen bonds, forming hybrid PNA-nucleic acid (DNA or RNA) duplexes. This system mimics the well-established antisense/antigene oligonucleotide strategy.

Overall, G-quadruplex targeting has been proposed as a novel therapeutic strategy for the treatment of various diseases, including cancer and infections.^1^^,^^177^^,^^178^ In this context, PNAs have emerged as powerful tools due to their high binding affinity, sequence specificity, and resistance to enzymatic degradation.^179^^,^^180^^,^^181^ PNAs can be designed to selectively recognize and bind G-quadruplex-forming sequences, thereby stabilizing or disrupting G4s. This capability enables precise modulation of gene expression and telomeric functions. In the early 2000s, the first reports on the use of PNAs to interrupt G-quadruplex formation appeared. Datta et al. described for the first time the use of short PNA oligomers to target a folded DNA G-quadruplex—the TBA.^182^ The authors showed the successful G-quadruplex invasion by PNA (7-mer) using circular dichroism spectroscopy and an organic dye indicator. They also investigated the effect of overhanging bases on the stability of PNA-DNA heteroduplex formation and found that the addition of the 4-base overhangs stabilizes the created duplex.^182^ Interestingly, Green et al. proposed an alternative method for detecting the disruption of G-quadruplex formation by PNA.^183^ In this study, the FRET technique was utilized to monitor the unfolding of human telomeric G4s in the presence of complementary PNA.^183^ A complementary PNA oligomer was used to trap the G-quadruplex during unfolding, allowing dynamics of the G4s and the complexity of its unfolding mechanism to be probed.^183^

Another example is provided by Sarkar and Armitage, who designed the gamma-modified peptide nucleic acid (γPNA) oligomers to target a G-rich sequence in the NS5 coding region of the West Nile virus (WNV) genome.^184^ Biophysical analyses confirmed that the RNA sequence forms a moderately stable G4 structure under physiological conditions. Moreover, three complementary γPNAs were shown to invade and bind to the target G4 structure with low femtomolar affinity at physiological temperature. This study underlines that γPNAs may represent a new approach for inhibiting both WNV replication and transcription, thereby representing a generally useful antiviral strategy.^184^

Although existing studies have shown that PNAs can disrupt G-quadruplexes, these examples reflect only a limited aspect of their broader utility. More advanced applications involve PNA conjugates, which provide enhanced specificity and offer improved cellular uptake, localization, or multifunctionality.^168^^,^^185^^,^^186^ The conjugation of PNAs to small molecules, peptides, or imaging agents enhances their potential as therapeutic or diagnostic compounds. Moreover, PNAs, which are chemically linked to other functional moieties, represent a more refined and targeted strategy. For instance, a study by Tassinari et al. demonstrated a novel approach based on naphthalene diimide (NDI)-PNA conjugates.^187^ This approach is schematically presented in Figure 6B, where ligand-PNA conjugates are designed for selective recognition and binding G-quadruplex structures. Using biophysical and biomolecular assays, the authors demonstrated that NDI-PNA conjugates can specifically interact with the target G4s within the HIV-1 LTR region, which contains overlapping and mutually exclusive G-quadruplex structures.^187^ Additionally, the presence of red-fluorescent emission and cellular localization signals enables the new conjugates to enter cells, with their localization easily confirmed by fluorescence imaging.^187^

In a recent study, Arrigo and co-workers demonstrated that PNA conjugates can target mRNA G-quadruplex structures to modulate gene expression *in vivo*.^188^ This study explored the stabilization of the RNA G-quadruplex within the 5′-UTR of *N*-RAS (proto-oncogene) using PNA1-heterocycle-PNA2 conjugates. Biophysical and biochemical analyses (CD, ITC, and *in vitro* translation assays) revealed that these conjugates bind selectively to the G-quadruplex and adjacent single-stranded RNA regions, effectively stabilizing the structure and influencing the expression of the oncogenic protein.^188^ The authors highlighted the therapeutic potential of PNA conjugates for gene regulation. By stabilizing the RNA G-quadruplex within the 5′-UTR of the *N*-RAS transcript, these conjugates suppress translation of oncogenic protein, providing a promising strategy for targeted cancer therapy.^188^

Recent advances show that PNA-based conjugates can dramatically increase the selectivity and functional potency of G4s recognition compared with small molecules alone. The conjugation strategies propose dual modes of action, structure binding (e.g., an NDI moiety) with sequence flank hybridization (PNA/LNA), and enable added functionality (photo-control, imaging tags, and cell-targeting peptides). These strategies have been demonstrated in defined promoter contexts (e.g., HIV-1 LTR)^187^ and in model RNA G4 systems. Currently, they are being integrated with advanced delivery platforms for cellular and *in vivo* applications, improving their potential for precise diagnostics and therapy.

A further strategy (C) for G4 targeting involves the use of homologous PNAs to form heteroquadruplexes with DNA or RNA G4 sequences (Figure 6C). This method uses the sequence specificity and high affinity of PNAs to modulate G4 folding and stability. This approach is conceptually similar to ASO-based strategies; however, PNAs offer enhanced chemical stability and greater resistance to nuclease degradation than ASOs. Despite this, PNA-based approaches are often limited by poor cellular uptake and therefore require specialized d8elivery strategies. For example, a study demonstrates that homologous PNAs can specifically recognize and form stable four-stranded hybrids with DNA G-quadruplexes.^189^ This interaction results in a well-defined PNA_2_-DNA_2_ structure with parallel-strand orientation and distinctive ion preferences.^189^ This finding highlights the potential for developing PNAs as a high-affinity, complementary strategy for targeting complex nucleic acid structures. On the other hand, PNA-based heteroquadruplex formation remains less explored in biological and *in vivo* contexts. For these reasons, this strategy is not discussed in detail in this review; nevertheless, it represents a complementary approach to G4 modulation, with distinct advantages and limitations.

While ligand-ASO and PNA-based conjugates provide exceptional specificity for G4 modulation, their transition from *in vitro* tools to *in vivo* therapeutics is primarily hindered by significant delivery challenges. A major limitation of ASOs remains their susceptibility to degradation and poor cellular uptake.^190^ This challenge is even more pronounced for PNA-based tools. Despite their high enzymatic stability and binding affinity, their neutral backbone leads to inherently low cell membrane permeability.^191^ To overcome these barriers, several delivery strategies have been integrated into conjugate design. Improving PNA uptake can also be achieved by the PNA chemical modifications, peptide conjugation (using cell-penetrating peptides [CPPs], which are frequently conjugated to PNAs),^192^ using prodrug strategies (such as the Mal2-G4-ASO platform),^4^ and targeted ligands (like anisamide, which enables receptor-mediated uptake by specifically recognizing proteins).^193^

Future perspectives for improving delivery efficiency involve the use of modern delivery platforms, such as lipid nanoparticles^192^ or viral vectors, which are currently being adapted for these G4-specific tools.^190^^,^^194^ Furthermore, the development of late-stage modification platforms, such as those based on phosphate triesters, enables the incorporation of multiple functional groups into a single conjugate. These include imaging tags and cell-targeting moieties.^158^ This multifunctionality of the phosphate probes is expected to refine the pharmacokinetics of G4-targeted agents, moving them closer to clinical viability. To conclude, Table 2 summarizes the currently described approaches for targeting G-quadruplexes, including small-molecule ligands, ASOs, ligand-ASO conjugates, and PNA-based tools. It outlines their principles of action as well as their main advantages and limitations.

**Table 2:** Comparison of molecular strategies for targeting and modulating G4 structures

	Small-molecule ligands	Antisense oligonucleotides (ASOs)	Ligand-ASO conjugates	Peptide nucleic acids (PNAs)
Principle of action	small aromatic molecules bind G4 structure mainly through π−π stacking with tetrads and additionally via groove, loop, or cation-π interactions, often stabilizing the G4	short nucleic acid strands bind the target sequences via Watson-Crick base pairing, enabling sequence-specific modulation of G4 formation, stabilization, or disruption	hybrid molecules combining a G4-binding ligand with ASO; the ASO provides sequence recognition, the ligand binds and stabilizes G4 structure	synthetic nucleic acid analogues with a neutral peptide-like backbone that hybridize with DNA/RNA via base pairing; can stabilize, disrupt G4s, or form heteroquadruplexes
Advantages	high structure specificity, simple chemical structure, often good cell permeability, strong G4 stabilization, some compounds in clinical trials	high sequence specificity – selective targeting of G4-forming sequences, adaptable design, versatile mechanisms of action (stabilization, destabilization, heteroduplex formation), approved ASO drugs	higher specificity than small molecules alone, stronger binding affinity via dual interaction, improved G4 stabilization, structure-specific recognition	very high binding affinity and sequence specificity, resistance to enzymatic degradation, improved hybridization stability through neutral backbone
Disadvantages	limited specificity (recognizes general topology rather than specific G4), off-target binding to other secondary structures, potential cellular toxicity	poor cellular uptake due to large size and negative charge, susceptibility to nuclease degradation, accessibility issues when G4 is folded, competition with RNA-binding proteins	limited *in vivo* validation, synthetic complexity, potential stability and delivery issues, pharmacokinetics not well characterized	limited *in vivo* validation, very poor cellular uptake, delivery systems required, more difficult synthesis than ASO, limited ability to invade pre-formed G4s

## Other strategies and future perspectives

Beyond the approaches described previously, several additional strategies have been developed to exploit conjugate-based systems for targeting G-quadruplex structures. These methods aim to selectively recognize and stabilize G4 motifs present, e.g., within viral genomes or regulatory regions, thereby interfering with essential steps of the viral life cycle. By integrating diverse molecular scaffolds and targeting moieties, these conjugates offer a versatile platform for modulating biological processes and gene expression through G4 recognition. The following section briefly discusses recent advances and representative examples of these conjugate-based strategies.

In 2024, Kwok and colleagues designed and synthesized an L-aptamer–ASO conjugate, L-Apt.4–1c-ASO15nt_(APP)_, targeting the amyloid precursor protein (APP) RNA G4 region. This new aptamer binds the RNA G4 structure, while the ASO hybridizes to flanking sequences, enabling highly selective recognition.^163^ The conjugate exhibits sub-nanomolar affinity, discriminates APP RNA G4 from other G4s, and inhibits APP protein expression *in vitro* and in cells. This study indicates that such a tool modulates gene expression by blocking DHX36-mediated unwinding, repressing translation, and inducing RNase H-dependent mRNA degradation, demonstrating its potential as a versatile tool for RNA G4-targeted gene regulation.^163^ This newly developed tool may be used to understand G4-mediated gene expression and gain deeper insights into potential treatments for diseases having molecular mechanisms associated with G4 structures.

An interesting study by Nadai et al. reported the development of a novel NDI-tetraaza cycloalkane conjugate.^195^ Inspired by the multitargeting strategy of current HIV-1 therapies, these conjugates were designed to simultaneously target multiple steps of the viral life cycle and potentially reduce the emergence of antiviral resistance.^195^ The NDI moiety binds and stabilizes LTR G-quadruplexes to inhibit promoter activity, while the tetraaza cycloalkane mimics AMD3100 (a small molecule) to block HIV-1 entry via the CXCR4 coreceptor. The NDI-metal-organic complexes exhibited enhanced G4 binding *in vitro* and dose-dependent inhibition of LTR activity and viral entry into cells. These novel tools simultaneously prevent viral entry via CXCR4 and inhibit LTR promoter activity by stabilizing the corresponding G-quadruplexes. This confirms that the conjugates act as G4-based HIV-1 inhibitors with a dual mode of action. The authors reported that integrating multiple targets into a single compound may both simplify treatment protocols and enhance antiviral efficacy, ultimately benefiting patient outcomes.^195^

Another example of a similar strategy is the modulation of DNA G-quadruplex structures by a small-molecule conjugate, reported by Mao and co-workers.^196^ In this study, the PDS compound was conjugated with a polyamide (PA) that selectively recognizes DNA sequences flanking the G4 region. Using optical tweezers, the binding of the PA-PDS conjugate to the human telomerase reverse transcriptase (hTERT) promoter revealed distinct intermediate structures corresponding to two G4 folding patterns. The authors found that PA promotes folding of a hairpin-G4 structure, while PDS facilitates the formation of two tandem G4s. Additionally, *in vitro* replication stop assays and *in vivo* dual-luciferase assays confirmed the effectiveness of new conjugates in targeting hTERT.^196^ This ligand-dependent folding approach may prove useful for developing drugs targeting hTERT and other oncogenes via the interactions with the G4s.

A further notable example of a G4-targeting approach was described by Qu and colleagues, who proposed metallosupramolecular complexes to target mpox virus (MPXV) RNA G-quadruplex and therefore enhance immune responses against the virus.^2^ Using an integrated bioinformatics, biophysical, and biological approach, they identified a highly conserved RNA G-quadruplex in the MPXV *A5L* mRNA (encoding 39-kDa virion core protein). Additionally, they revealed that modulation of this G4 stability, either by chiral metallosupramolecular complexes or the helicase DHX36, affects the A5L protein expression. Notably, the MH3 Λ complex selectively binds the viral RNA G4 without interacting with human DNA G4s, highlighting its potential as a targeted therapeutic for mpox virus.^2^ Because the *A5L* gene is essential for immune activation and virion maturation, targeting G-quadruplex within MPXV *A5L* mRNA by MH3 Λ complex shows significant antiviral activity through immune enhancement.

An alternative method employs aptamers, oligonucleotides that adopt various structures (including G-quadruplexes), as antiviral agents. Therefore, this approach is based on the application of G4s as the targeting agents rather than targets. Such molecules can target viral proteins, for example, several HIV proteins, ultimately reducing viral infectivity.^197^ One example of G-rich oligonucleotides functioning as anti-HIV agents is the HIV-1 integrase inhibitor that adopts a six-layer G-quadruplex structure.^198^ Importantly, G4 aptamers can retain structural stability within cells even without chemical modifications. Beyond targeting HIV proteins, they may reduce respiratory syncytial virus infection.^199^ Additionally, a G4 aptamer has been identified that binds to the influenza A virus hemagglutinin in different viral strains.^87^

The innovative CRISPR-guided strategy enables targeted modulation of G4 structures at defined genomic loci.^200^^,^^201^ This tool is based on binding G4-stabilizing proteins or small-molecule ligands to catalytically inactive Cas9 (dCas9). This approach, termed ATENA (Approach to Target Exact Nucleic Acid alternative structures), enables programmable positioning of G4 binders near selected G4-forming sequences via guide RNAs.^201^ Thus, it promotes or stabilizes G4 folding in a locus-specific manner within living cells. By providing both genomic and intra-G4 selectivity, this platform addresses the poor specificity of conventional G4 ligands and enables functional analysis of individual G4s with minimal off-target effects.^201^ ATENA provides a versatile framework to modulate transcriptional and cellular processes driven by G4 structures. It also serves as a powerful tool for understanding processes regulated by G-quadruplexes and developing targeted G4-based therapeutic strategies. The ATENA strategy enables precise targeting of G-quadruplexes at specific loci. However, its use is limited by challenges in delivering dCas9, chromatin accessibility, and potential indirect cellular effects resulting from prolonged G4 stabilization.^201^

To conclude, recent developments in conjugate-based strategies have expanded the tools for selective targeting and stabilization of G-quadruplex structures. The examples discussed above illustrate how combining diverse molecular components can help to achieve precise modulation of G4 folding, gene expression, or viral replication.

## Conclusion

G-quadruplexes have emerged as critical regulatory elements in both cellular and viral genomes, influencing transcription, translation, and replication. Targeting these structures offers a unique approach for modulating gene expression and developing novel therapeutics. Nonetheless, a significant limitation in current research is the G4-ligand’s low selectivity for G-quadruplexes over duplexes, which can cause off-target effects. This emphasizes the necessity of highly selective and improved tools for targeting G-quadruplexes. Strategies such as ASOs, ASO conjugates, PNAs, PNA conjugates, and other innovative approaches, including small molecules, metallosupramolecular complexes, and ligand-guided oligonucleotides, demonstrate remarkable potential for achieving sequence- and structure-specific recognition of G4s. By stabilizing or modulating G4 formation, these tools can precisely control gene expression, inhibit viral replication, and potentially treat G4-related diseases.

While significant progress has already been made, future efforts should focus on improving the selectivity, stability, and delivery efficiency of different tools targeting G4s to enhance their therapeutic potential. Advances in structural biology, computational modeling, and rational design will further facilitate the development of next-generation G4-targeted agents with broad applicability, e.g., in antiviral or anticancer therapies.

## Acknowledgments

We thank all the researchers who have advanced the field and apologize to those whose work was not cited due to space constraints. This research was funded by the National Science Centre, grant number: UMO-2023/49/B/ST4/03763 (M. Szabat).

Figures were created using the BioRender application (https://BioRender.com) and CorelDRAW software.

## Author contributions

M. Sokulska, M.N., T.C., M.R., and M. Szabat, writing – original draft; M. Szabat, conceptualization, funding acquisition, and supervision. All authors have read and agreed to the published version of the manuscript.

## Declaration of interests

The authors declare no conflict of interest. The funders had no role in the design of the study, in the collection, analyses, or interpretation of data, in the writing of the manuscript, or in the decision to publish the results.

## Declaration of generative AI and AI-assisted technologies in the writing process

During the preparation of this work, the author(s) utilized OpenAI’s ChatGPT and Grammarly to refine, paraphrase, and verify grammar, thereby enhancing clarity and fluency. However, the core ideas, structure, and content were developed by all co-authors in consultation with M. Szabat. After employing these tools, the author(s) reviewed and edited the content as necessary and take full responsibility for the publication’s content.
